# Addictive behaviours in the employees of an electricity company in Tunisia

**DOI:** 10.1192/j.eurpsy.2023.1392

**Published:** 2023-07-19

**Authors:** M. A. Ghrab, I. Sellami, A. Abbes, M. Hajjaji, K. Jmal Hammami, M. L. Masmoudi

**Affiliations:** 1 Occupational medicine, University Hospital Hedi Chaker, Sfax, Tunisia; 2 University of Sfax, Sfax, Tunisia

## Abstract

**Introduction:**

In Tunisia, tobacco control remains one of the main country’s health strategies. However, it seems that the scourge of tobacco is still a prevalent problem and it’s often associated with other addictive behaviours like alcohol use.

**Objectives:**

Evaluate the addictive behaviours of the employees of an Electricity company in Tunisia and their impact on their mental health.

**Methods:**

We conducted a cross-sectional study in May 2022. A pre-established questionnaire was filled out during a sensitization campaign that took place in the company. We used the Fagerstörm test and the AUDIT questionnaire to evaluate tobacco and alcohol dependency respectively. Signs of depression and anxiety were evaluated by the Hospital Anxiety and Depression scale (HAD). Collected data were analyzed using IBM SPSS statistics version 23.0.

**Results:**

Our population consisted of 83 employees. The average age was 40.79±11.23. Males represented 65.1% of employees. The mean of seniority was 15.23±10.82 years. Forty-one per cent were overweight and 22.9% had obesity. The mean Body Mass Index (BMI) was 27.17±3.92. Twenty-five employees (30.1%) were active smokers and 7 (8.5%) consumed alcohol. All of them were males. The nicotine dependency test’s mean was 4.12±2.78 and 28% of smokers had a high to a very high nicotine dependency. The mean score of the AUDIT questionnaire was 9.71±10.76. Four alcohol consumers (57.1%) had harmful alcohol use and 2 of them (28.5%) had alcohol dependency. The evaluation of the HAD score showed that the mean anxiety score was 7.59±3.13 and the mean depression score was 6.44±3.71. Twelve per cent and 10.8% of employees had elevated anxiety and depression scores respectively. Bivariate analysis showed that depression is significantly associated with the female sex (p=0.023) and with a lower number of service years (p=0.019). Anxiety was significantly associated with a high BMI (p=0.027). Anxiety and depression were not associated with alcohol or tobacco consumption.

**Conclusions:**

Smoking and drinking are common in our society. Sensibilization campaigns must focus on motivating workers to quit those addictive behaviours and promote a healthier lifestyle.

**Disclosure of Interest:**

None Declared

